# Macrosystems EDDIE teaching modules significantly increase ecology students' proficiency and confidence working with ecosystem models and use of systems thinking

**DOI:** 10.1002/ece3.6757

**Published:** 2020-10-02

**Authors:** Cayelan C. Carey, Kaitlin J. Farrell, Alexandria G. Hounshell, Kristin O'Connell

**Affiliations:** ^1^ Department of Biological Sciences Virginia Tech Blacksburg VA USA; ^2^ Odum School of Ecology University of Georgia Athens GA USA; ^3^ Science Education Resource Center Carleton College Northfield MN USA

**Keywords:** active learning, ecology education, macrosystems EDDIE, simulation modeling, teaching modules, undergraduate curricula

## Abstract

Simulation models are increasingly used by ecologists to study complex, ecosystem‐scale phenomena, but integrating ecosystem simulation modeling into ecology undergraduate and graduate curricula remains rare. Engaging ecology students with ecosystem simulation models may enable students to conduct hypothesis‐driven scientific inquiry while also promoting their use of systems thinking, but it remains unknown how using hands‐on modeling activities in the classroom affects student learning. Here, we developed short (3‐hr) teaching modules as part of the Macrosystems EDDIE (Environmental Data‐Driven Inquiry & Exploration) program that engage students with hands‐on ecosystem modeling in the R statistical environment. We embedded the modules into in‐person ecology courses at 17 colleges and universities and assessed student perceptions of their proficiency and confidence before and after working with models. Across all 277 undergraduate and graduate students who participated in our study, completing one Macrosystems EDDIE teaching module significantly increased students' self‐reported proficiency, confidence, and likely future use of simulation models, as well as their perceived knowledge of ecosystem simulation models. Further, students were significantly more likely to describe that an important benefit of ecosystem models was their “ease of use” after completing a module. Interestingly, students were significantly more likely to provide evidence of systems thinking in their assessment responses about the benefits of ecosystem models after completing a module, suggesting that these hands‐on ecosystem modeling activities may increase students’ awareness of how individual components interact to affect system‐level dynamics. Overall, Macrosystems EDDIE modules help students gain confidence in their ability to use ecosystem models and provide a useful method for ecology educators to introduce undergraduate and graduate students to ecosystem simulation modeling using in‐person, hybrid, or virtual modes of instruction.

## Graphical abstract

Macrosystems EDDIE modules are able to provide students a realistic introduction to ecosystem modeling and an appreciation of the power of simulation models for exploring ecosystem dynamics. After completing a 3‐hr ecosystem modeling activity, the proportion of ecology students reporting potential benefits of ecosystem models ‐ including (a) cost savings, time savings, ease of use, and model setup/manipulation ‐ and potential challenges of ecosystem models ‐ including (b) programming/coding ‐ significantly increased. Stars indicate statistical significance; ***p < .001; **p < .01; *p < .05.

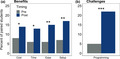

## 1 INTRODUCTION

Given the scale of environmental problems facing society, including climate change, land use change, and species invasions, it is critical that ecology students receive training in systems thinking to help prepare them to tackle complex ecosystem‐scale challenges (Hogan & Weathers, 2003; Weathers et al., 2016). Systems thinking—here, defined as the ability to recognize how interrelationships between individual components of a system affect the overall system function, which may include nonlinear dynamics, feedback loops, and/or time delays among components (Arnold & Wade, 2015)—is a skill increasingly highlighted as necessary to solve complex social and ecological problems (Bergan‐Roller, Galt, Chizinski, Helikar, & Dauer, 2018; Gilbert, Gross, & Kreutz, 2019). Paralleling the emergence of macrosystems ecology as a subdiscipline within the larger field of ecology (Fei, Guo, & Potter, 2016), ecology students increasingly need to understand how ecological phenomena occur over different spatial and temporal scales; how ecological processes may include multiple feedback loops and time lags; and how populations, communities, and ecosystems may interact nonlinearly over space and time (Dodds & Whiles, 2019; Heffernan et al., 2014). Simultaneously, ecology students need to learn new computational and data science skills that are emerging as necessary tools for a growing number of jobs across every sector (Durden, Luo, Alexander, Flanagan, & Grossmann, 2017; Michener & Jones, 2012).

One approach for simultaneously teaching ecology students about ecosystem‐scale dynamics and computational skills while potentially increasing their use of systems thinking is to embed hands‐on ecosystem simulation modeling activities into undergraduate and graduate curricula. Ecosystem simulation models harness the power of computers to improve our understanding of ecosystem‐scale behavior by enabling users to both simulate historical dynamics and make predictions about future conditions (Canham, Cole, & Lauenroth, 2004; Wiegert, 1975). While ecosystem simulation models are inherently simplified relative to natural ecosystems, they are increasingly used by ecologists to explore how ecosystems may change under different climate and land use scenarios (e.g., Asch, Pilcher, Rivero‐Calle, & Holding, 2016; Hipsey et al., 2019; Morales‐Marín, Rokaya, Sanyal, Sereda, & Lindenschmidt, 2019). In addition to serving as an important research tool, ecosystem simulation models can also be used for educational purposes and provide a practical way for students to conduct ecosystem‐scale scientific inquiry and practice systems thinking in a classroom setting (Gilbert & Justí, 2016). For example, students can make a priori hypotheses about how an ecosystem and its components will respond to a disturbance such as climate or land use change, develop model scenarios to test their hypotheses, and then see if their hypotheses are supported (following Assaraf & Orion, 2005; Gilbert et al., 2019).

Despite the potential benefits of using models as a teaching tool, simulation ecosystem modeling activities are rare in ecology curricula (Cottingham, Fey, Fritschie, & Trout‐Haney, 2017; Weathers et al., 2016). This may be because instructors and/or their students are intimidated by the idea of working with models (Anderson, McKenzie, Wellman, Brown, & Vrbsky, 2011; Valle & Berdanier, 2012), and thus it remains largely unknown how ecology students perceive the benefits and challenges of working with ecosystem models. If students have low confidence in their modeling abilities, this mental barrier may create a positive feedback in which they are less likely to attempt to use models in the future (Farrell & Carey, 2018). Conversely, if students gain confidence in their ability to use ecosystem models, they may be more interested and likely to continue using these tools long term (Farrell & Carey, 2018). It is also expected that students’ perceptions of the benefits and challenges of ecosystem models may become more realistic after working with a model, especially if they had no prior modeling experience.

To address these predictions, we developed three teaching modules with hands‐on ecosystem modeling activities as part of the Macrosystems EDDIE (Environmental Data‐Driven Inquiry & Exploration) program. Macrosystems EDDIE is a U.S. National Science Foundation‐supported initiative to develop the first comprehensive undergraduate‐focused curriculum in macrosystems ecology while fostering students’ computational literacy and quantitative skills (Farrell & Carey, 2018). Each of the Macrosystems EDDIE modules focuses on a fundamental macrosystems concept (e.g., cross‐scale interactions and teleconnections) through the lens of limnology, with an emphasis on teaching students how ecological phenomena interact across multiple spatial and temporal scales.

We embedded the Macrosystems EDDIE modules into ecology curricula at multiple educational institutions and assessed student perceptions of ecosystem simulation models. Specifically, we examined if a 3‐hr modeling activity could increase ecology students’ self‐reported proficiency and confidence working with models and their use of systems thinking skills. We used pre‐ and postmodule surveys to address two questions related to the efficacy of the modules: (1) How do hands‐on Macrosystems EDDIE modeling activities in the classroom affect students' proficiency with, and perceptions of, ecosystem models? and (2) How does using hands‐on Macrosystems EDDIE modeling activities affect students' use of systems thinking? The modules were tested in this study using an in‐person mode of instruction but could be readily adapted for virtual or hybrid modes (see Section 2.3 below).

## 2 MATERIALS AND METHODS

### 2.1 Module overview and framework

Each Macrosystems EDDIE module taught in this study introduces students to ecosystem simulation modeling of lakes in the R statistical environment. Students choose which lakes they want to model and then complete scaffolded modeling activities that build from simple to more complex, based on the pedagogy of the 5E learning cycle (engagement, exploration, explanation, expansion, and evaluation; Bybee et al., 2006, Table 1) adapted for Project EDDIE teaching modules (ProjectEDDIE.org; Carey, Gougis, Klug, O'Reilly, & Richardson, 2015; O'Reilly et al., 2017). The flexible structure of the Macrosystems EDDIE modules enables instructors to teach the activities that are most appropriate for their course, instruction mode, and student experience level, as some module activities can be completed in a 1‐hr lecture session, whereas an entire module can be taught in a 3‐hr laboratory session.

**TABLE 1:** Phases of the 5E learning cycle and a description of how the phases were incorporated into each Macrosystems EDDIE module's activities

5E Phase	5E Phase Purpose	Module 1: Climate change effects on lake temperatures (Carey et al., 2018)	Module 2: Cross‐scale interactions (Carey & Farrell, 2019)	Module 3: Teleconnections (Farrell & Carey, 2019)
Engagement	Introduce topic, gauge students’ preconceptions, call up students’ schemata	Short introductory lecture	Short introductory lecture	Short introductory lecture
Exploration	Engage students in inquiry, scientific discourse, evidence‐based reasoning	Development of hypotheses of how climate change affects lakes; testing of these hypotheses by forcing lake models with climate scenarios to see how the lakes respond	Development of hypotheses of how climate change and land use affect lakes; testing of these hypotheses by forcing lake models with climate and land use scenarios to see how the lakes respond	Development of hypotheses of how teleconnections affect lakes; testing hypotheses by forcing lake models with El Niño scenarios to see how different lakes respond
Explanation	Engage students in scientific discourse, evidence‐based reasoning	In‐class discussion of the effects of the different climate scenarios	In‐class discussion of the effects of the different climate and land use scenarios	In‐class discussion of the different effects of the El Niño Southern Oscillation on lake temperatures and ice cover in lakes from different regions
Expansion	Broaden students’ schemata to account for more observations	Using the GRAPLEr software to create hundreds of different climate scenarios	Assessing cross‐scale interactions by comparing combined climate + land use scenarios to separate climate and land use model output; comparing how multiple lakes respond to the same scenarios	Assessing teleconnections by comparing how lakes from different regions respond to the same environmental phenomenon; predicting how lakes in other regions would respond to El Niño events
Evaluation	Evaluate students’ understanding, using formative and summative assessments	In‐class discussion of how climate change can affect lake thermal structure	In‐class discussion of how climate change and land use change can interact to affect phytoplankton blooms in lakes	In‐class discussion of how teleconnections can affect water temperatures and ice cover in lakes

Each Macrosystems EDDIE module consists of an annotated instructor's manual, premodule readings, Microsoft Word student handout, R code for running the predeveloped ecosystem simulation models set up for each lake, short Microsoft PowerPoint lecture for the instructors to introduce the learning objectives of the module, and postmodule discussion questions. By using Microsoft Word, Microsoft PowerPoint, and R software, our goal was to develop module materials that could easily be modified by instructors to fit the needs of their students and curriculum. All teaching materials are publicly available at www.MacrosystemsEDDIE.org and are also published in the Environmental Data Initiative (EDI) repository with digital object identifiers (DOIs) for versioning control (Carey, Aditya, Subratie, Figueiredo, & Farrell, 2018; Carey & Farrell, 2019; Farrell & Carey, 2019).

We specifically developed modules that introduce students to authentic ecosystem simulation modeling using realistic, hands‐on activities. For example, rather than having students “point and click” through simplified modeling exercises, they have to modify predeveloped R code to alter parameter values and driver datasets to model actual lakes, using data observed by high‐frequency sensors in each ecosystem. In each of the modules, students are taught fundamental modeling concepts, such as ecosystem model dimension, the role of parameters and variables in model equations, and how different driver datasets can be used as inputs to run different model scenarios.

### 2.2 Module descriptions

Three Macrosystems EDDIE modules were assessed in this study (Table 1): Climate Change Effects on Lake Temperatures (Module 1, Carey et al., 2018), Cross‐Scale Interactions (Module 2, Carey & Farrell, 2019), and Teleconnections (Module 3, Farrell & Carey, 2019). In the Climate Change Effects on Lake Temperatures module, students learn how to set up a lake ecosystem simulation model and "force" the model with climate scenarios of their own design to test hypotheses about how the lake may change in the future (Carey et al., 2018). Once students have mastered running one climate scenario for their lake, they learn how to use a distributed computing tool to scale up and run hundreds of different climate scenarios for their lake (Carey et al., 2018). In the Cross‐Scale Interactions module, students learn how to set up an ecosystem simulation model for a GLEON (Global Lake Ecological Observatory Network) lake of their choice and “force” the model with climate and land use scenarios (e.g., a 2°C increase in air temperatures, a doubling of phosphorus inputs) to test hypotheses about how local and regional drivers interact to promote or suppress phytoplankton blooms in different lakes (Carey & Farrell, 2019). In the Teleconnections module, students learn how to set up an ecosystem simulation model for a GLEON or NEON (National Ecological Observatory Network) lake of their choice and “force” the model with El Niño climate scenarios to test hypotheses about how global drivers interact with regional weather and local lake characteristics to affect lake temperatures and ice cover (Farrell & Carey, 2019). A description of how each module's activities fulfills the objectives of the 5E learning cycle is given in Table 1.

All three of these Macrosystems EDDIE modules use the General Lake Model (GLM), a flexible, open‐source numerical simulation model (Hipsey et al., 2019). GLM simulates vertically resolved thermal layers in response to meteorological and inflow driver data, and can easily be coupled to Aquatic EcoDynamics modules to simulate oxygen dynamics, biogeochemical cycling, and plankton food webs (Hipsey, Bruce, & Hamilton, 2013). Given its rapidly growing user base (e.g., Bruce et al., 2018), integration with R software (e.g., Read, Gries, & Read, 2016; Winslow et al., 2016), and quickly expanding set of tools for model manipulation and visualization (e.g., Bueche et al., 2020), GLM is a widely used ecosystem simulation model within the aquatic research community.

### 2.3 Remote module instruction

The Macrosystems EDDIE modules were developed for in‐person instruction, but could be easily converted for synchronous or asynchronous remote instruction. These modifications could include running the modules using RStudio Cloud software, which would obviate the need for students to install R software on their own computers. RStudio Cloud can streamline programming instruction and enables instructors to review students' R code remotely. In a remote learning environment, the students would complete the premodule readings on their own. The instructor would either livestream the introductory PowerPoint lecture for synchronous instruction or record it for asynchronous viewing. The student handout could be integrated into an online assignment via the instructor's remote learning management system, so that instructors could provide feedback and/or grade the student responses. The instructor could supplement these self‐guided activities with virtual office hours to answer student questions or troubleshoot code via RStudio Cloud software, or moderate a postmodule discussion using predeveloped questions. While RStudio Cloud has a fee for use, it provides a useful option for teaching the Macrosystems EDDIE curriculum remotely by enabling hands‐on R activities without requiring extensive software installations that can challenge novice programmers. Accessing a within‐institution server that runs RStudio Server Pro could be another option for including these types of modeling activities in remote classrooms without using RStudio Cloud.

### 2.4 Study design and student assessment

We assessed the efficacy of the three Macrosystems EDDIE modules in 21 ecology classrooms across 17 colleges and universities that spanned a range of institution types and a conference workshop (Table 2). All students who participated in this study completed an online pre‐ and postmodule assessment. The modules were taught using face‐to‐face instruction in primarily upper‐level (3rd‐ and 4th‐year) undergraduate courses (13 classrooms), but also included three introductory undergraduate classrooms, three mixed upper‐level undergraduate/graduate classrooms, two graduate classrooms, and a conference workshop, ranging from *n* = 3 to *n* = 39 consenting students per classroom. Faculty volunteers were recruited for this study via presentations on Macrosystems EDDIE at scientific conferences and through email lists and social media. Because the number of students, course type and content, module taught, and student experience level varied substantially among classrooms and were not controlled for in our study design, analyses focused on the total population of students who consented to participate in this study (*n* = 277), not individual classrooms or modules.

**TABLE 2:** Classrooms assessed by institution type

Institution type	Number of classrooms	Student participants	Number of classrooms testing each module		
			Module 1	Module 2	Module 3
Associate's College	1	8	1	0	0
Baccalaureate Colleges	2	3–13	1	1	0
Master's Colleges and Universities	3	3–13	1	2	0
Doctoral Universities^a^	15	3–39	4	8	3
Conference Workshop	1	9	0	0	1

Note

Student participants indicate the number of students who consented to the use of their data in this study.

^a^ Includes 2 classrooms from universities outside the United States.

The Science Education Resource Center (SERC) at Carleton College provided independent educational evaluation of this project. SERC staff developed assessment questions, language, and scales and administered the assessment via their online platform. The pre‐ and postmodule assessments were identical surveys that included multiple‐choice items about the students’ perceptions and confidence of their computational and modeling skills and their perceived knowledge of ecosystem simulation modeling (see Table S1 for survey instrument). For each multiple‐choice question, students responded using a Likert scale from 1 (low) to 5 (high; Table S1). The multiple‐choice items were complemented by two free‐response short answer questions on the benefits and challenges of using ecosystem models (Table S1), which were used to assess student perceptions of ecosystem simulation models and their use of systems thinking. Premodule assessments were completed within 10 days prior to the module being taught, while postmodule assessments were completed between 1 and 14 days after the module was taught. We designed the assessments to be completed within ~15 min to avoid survey fatigue. Only student participants who voluntarily consented to the use of their data were included in our study following our Institutional Review Board protocol (Carleton College IRB #0002470).

### 2.5 Data analyses

We first tested for changes in student perceptions of their proficiency, confidence, and likely future use of simulation modeling and the General Lake Model using Wilcoxon signed‐rank tests of paired student responses from the pre‐ and postmodule assessment multiple‐choice questions. We used this nonparametric test because of the ordinal nature of the Likert data. This analysis was based on students’ first use of a Macrosystems EDDIE module (*n* = 173 students who completed both pre‐ and postmodule assessment questions; students who did not complete both the pre‐ and postmodule questions were excluded from the analysis). For each student, we calculated changes in reported proficiency, confidence, and likely future use for simulation modeling and the General Lake Model as the difference between the post‐ and preassessment Likert scores. *p*‐values were based on a normal approximation, with statistical significance set a priori at *α* = 0.05. Effect sizes were calculated as Z/√n separately for each metric (proficiency, confidence, likely future use) and assessment item (simulation modeling and the General Lake Model). We repeated these analyses for an additional multiple‐choice question on the students’ self‐reported knowledge of ecosystem simulation modeling. All analyses were conducted using R 3.6.1 (R Core Team, 2019).

The qualitative responses to the two assessment questions related to perceived benefits and challenges of ecosystem models were coded in two phases using a provisional coding method, beginning with an a priori list of themes and refined with themes which emerged from student responses (Miles, Huberman, & Saldana, 2020). Phase I focused on developing and refining coding criteria to enable consistent coding while also identifying useful themes in the responses (see Appendix S1 for detailed methods and Table S2 for the codebook). Phase II focused on applying the coding to the full set of pre‐ and postassessment student responses (from the subset of students who provided both pre‐ and postmodule responses). The result of this two‐phase process was a database of student responses to the two qualitative questions coded by the presence or absence of each of the themes in their responses (Table S2). Wilcoxon signed‐rank tests of paired student pre‐ and postmodule responses were then conducted for each of the themes as described above.

Finally, the same responses to the two qualitative assessment questions were reviewed for evidence of systems thinking (Appendix S1). The two evaluators independently coded student responses using an a priori codebook based on the four rubric categories developed by Iverson et al. (2019). These categories were as follows: (a) Student correctly identifies and describes a real‐world system including its parts; (b) Student correctly describes how a change in one part of the system, in turn, alters other parts of the system; (c) Student correctly explains how parts of the system interact using systems concepts such as feedbacks, equilibrium, rates, etc.; and (d) Student describes how an effect can be influenced by multiple causal factors. We supplemented the rubric with an additional category to capture evidence of systems thinking: (e) Student correctly describes that many variables may contribute to a given outcome or new aspects introduced in a system may influence predictions. The final database of student responses included the five individual categories as well as a collapsed category that summed all of the individual categories, indicating the presence or absence of any evidence of systems thinking (Table S3).

## 3 RESULTS

### 3.1 Question 1: How do hands‐on Macrosystems EDDIE modeling activities in the classroom affect students’ proficiency with, and perceptions of, ecosystem models?

#### 3.1.1 Students' perceived proficiency with models

**FIGURE 1:**
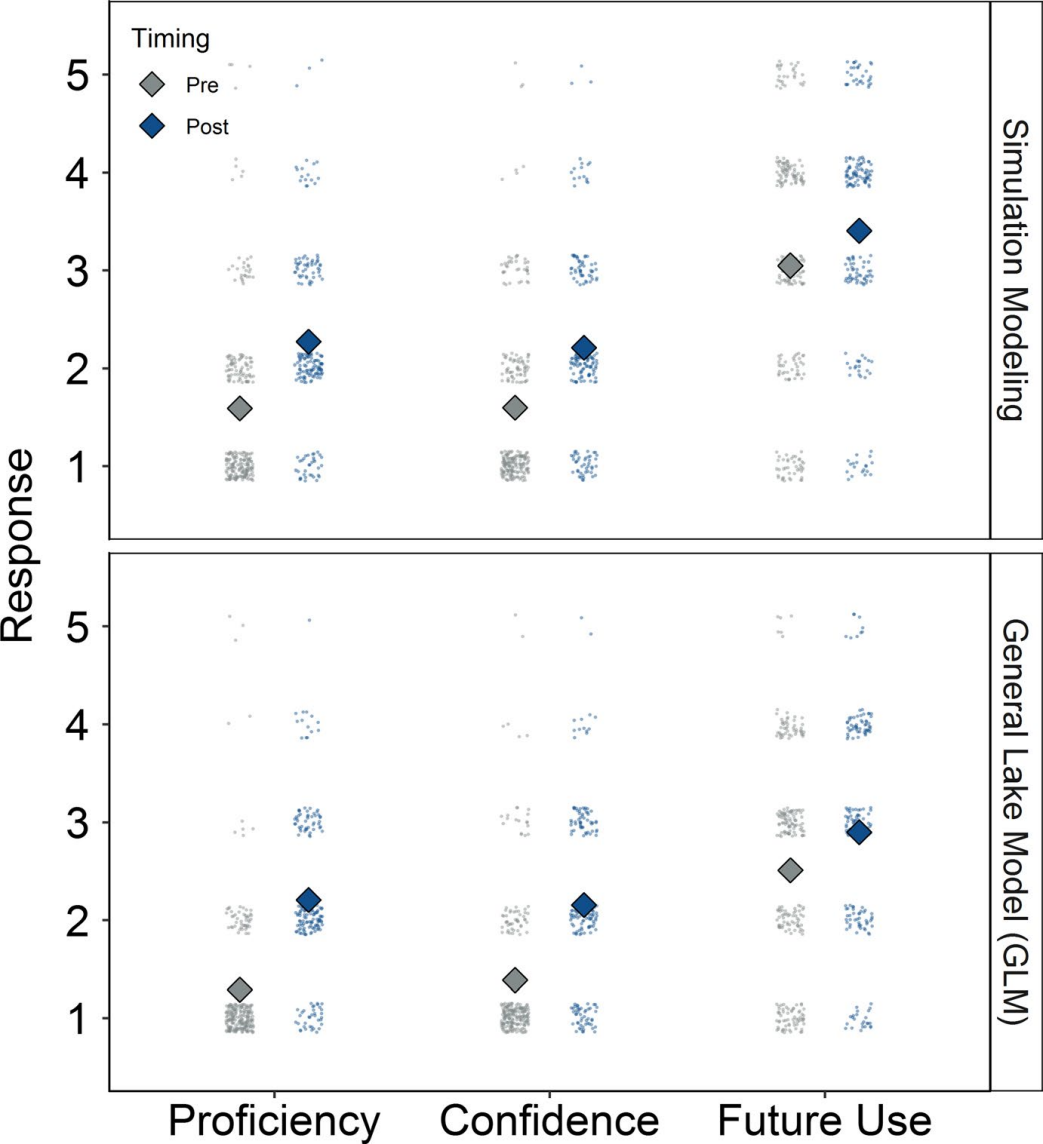
Student self‐reported proficiency, confidence, and likely future use of simulation modeling (a) and the General Lake Model (b) during premodule (gray) and postmodule (blue) assessments of their use of a Macrosystems EDDIE module. Questions used a Likert scale from 1 (low) to 5 (high). Jittered points represent individual students; diamonds denote mean among‐student responses

Across all students in our study, completing one Macrosystems EDDIE module significantly increased students’ self‐reported proficiency, confidence, and likely future use of simulation models and the General Lake Model (Table 3, Figure 1). On average, students' self‐reported proficiency responses changed from “basic proficiency” to “intermediate proficiency,” and their confidence responses changed from “somewhat confident” to “moderately confident” for both simulation modeling and the General Lake Model. Larger mean gains were observed in the students’ self‐reported proficiency, confidence, and likely future use of the General Lake Model than simulation modeling (Table 3), potentially because the General Lake Model metrics were lower on average than simulation modeling in the premodule assessment data (Figure 1).

**TABLE 3:** Differences in student self‐reported proficiency, confidence, and likely future use of simulation modeling and the General Lake Model (GLM), and their current knowledge of ecosystem models between premodule and postmodule assessments

Metric	Test statistic	Two‐tailed *p‐*value	*n*	Premodule mean (±1 *SE*)	Postmodule mean (±1 *SE*)	Effect size
Simulation modeling						
Proficiency	540	**<.001**	173	1.59 ± 0.05	2.27 ± 0.06	0.65
Confidence	757	**<.001**	173	1.60 ± 0.05	2.21 ± 0.07	0.61
Likely use	1,451	**.002**	173	3.05 ± 0.08	3.40 ± 0.08	0.24
GLM model						
Proficiency	267	**<.001**	172	1.29 ± 0.04	2.21 ± 0.06	0.73
Confidence	326	**<.001**	172	1.39 ± 0.05	2.16 ± 0.07	0.67
Likely use	1,442	**.001**	173	2.51 ± 0.07	2.90 ± 0.08	0.26
Knowledge of ecosystem models	446	**<.001**	172	2.17 ± 0.05	3.11 ± 0.05	0.72

Note

Test statistics and *p*‐values are for paired, two‐sided Wilcoxon signed‐rank tests. Questions used a Likert scale from 1 (low) to 5 (high).

**FIGURE 2:**
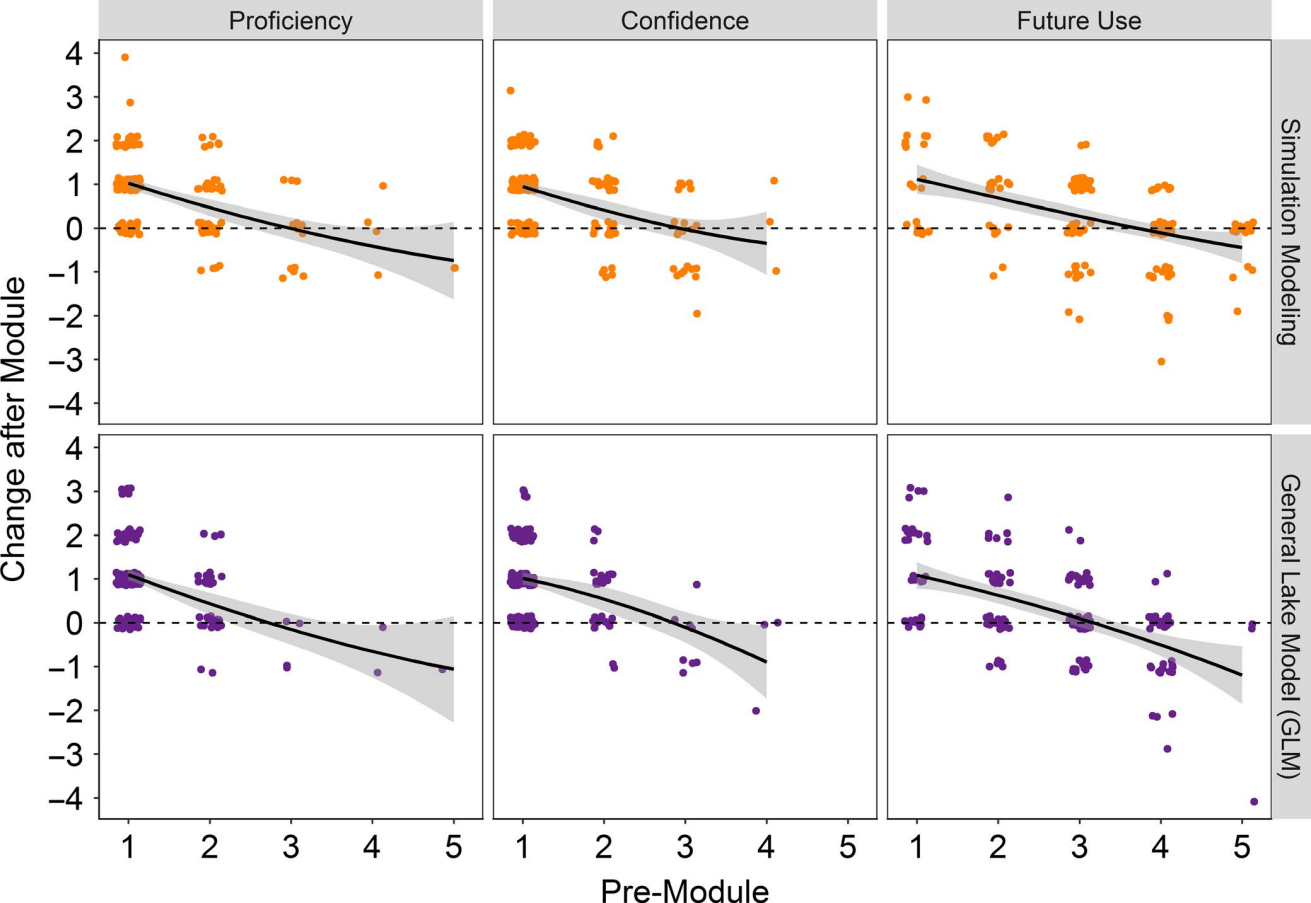
Students with the lowest premodule assessment responses on a Likert scale from 1 (low) to 5 (high) exhibited the greatest gains in self‐reported proficiency, confidence, and future use of simulation modeling and the General Lake Model (GLM) after completing a Macrosystems EDDIE module

A consistent pattern in the simulation modeling and General Lake Model results is that the greatest gains in students’ self‐reported proficiency, confidence, and likely future use were experienced by the students with the lowest premodule Likert scores (Figure 2). The students who reported “no proficiency” with modeling in the premodule survey generally responded in the postmodule survey that they now had “basic proficiency” or “intermediate proficiency.” In comparison, the students who reported initially that they had “advanced proficiency” or “expert proficiency” generally exhibited zero or negative gains, suggesting overconfidence in their initial self‐assessment. This pattern consistently emerged for both simulation modeling and General Lake Model proficiency, confidence, and likely future use (Figure 2).

**FIGURE 3:**
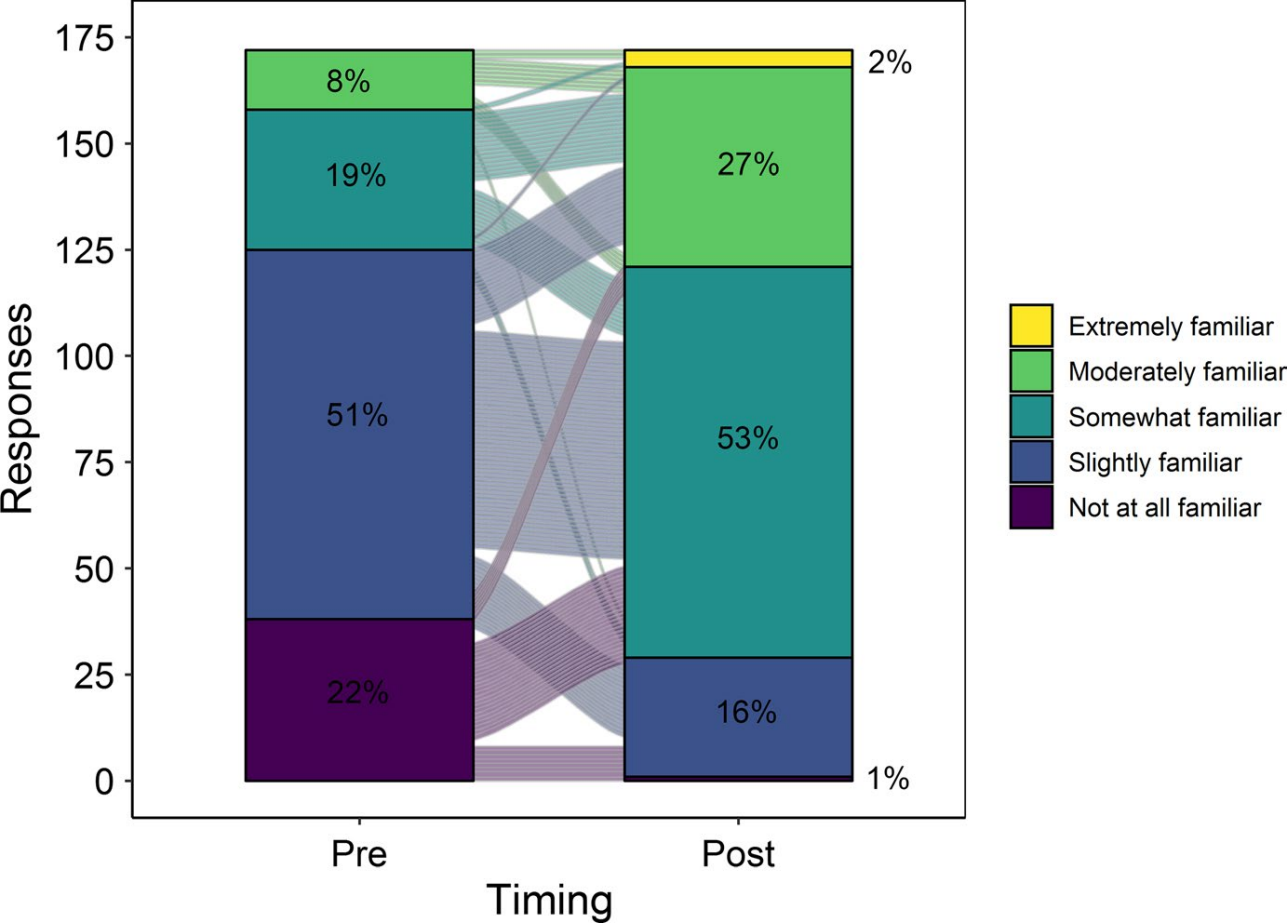
Changes in student self‐reported knowledge of ecosystem simulation modeling before and after using a Macrosystems EDDIE module. Percent of responses at each level of familiarity are shown for paired students in pre‐ and postmodule assessments

Overall, students reported significantly greater perceived knowledge of ecosystem simulation models after completing a Macrosystems EDDIE module (*p* < .001; Table 3, Figure 3). Prior to completing a module, students reported that their current knowledge of ecosystem simulation modeling on average was “Slightly familiar, I have heard of ecosystem simulation modeling, but cannot elaborate.” After completing a module, students on average reported that their perceived knowledge level on average was “Somewhat familiar, I could explain a little about ecosystem simulation modeling.”

### 3.2 Students' perceptions of model benefits and challenges

**FIGURE 4:**
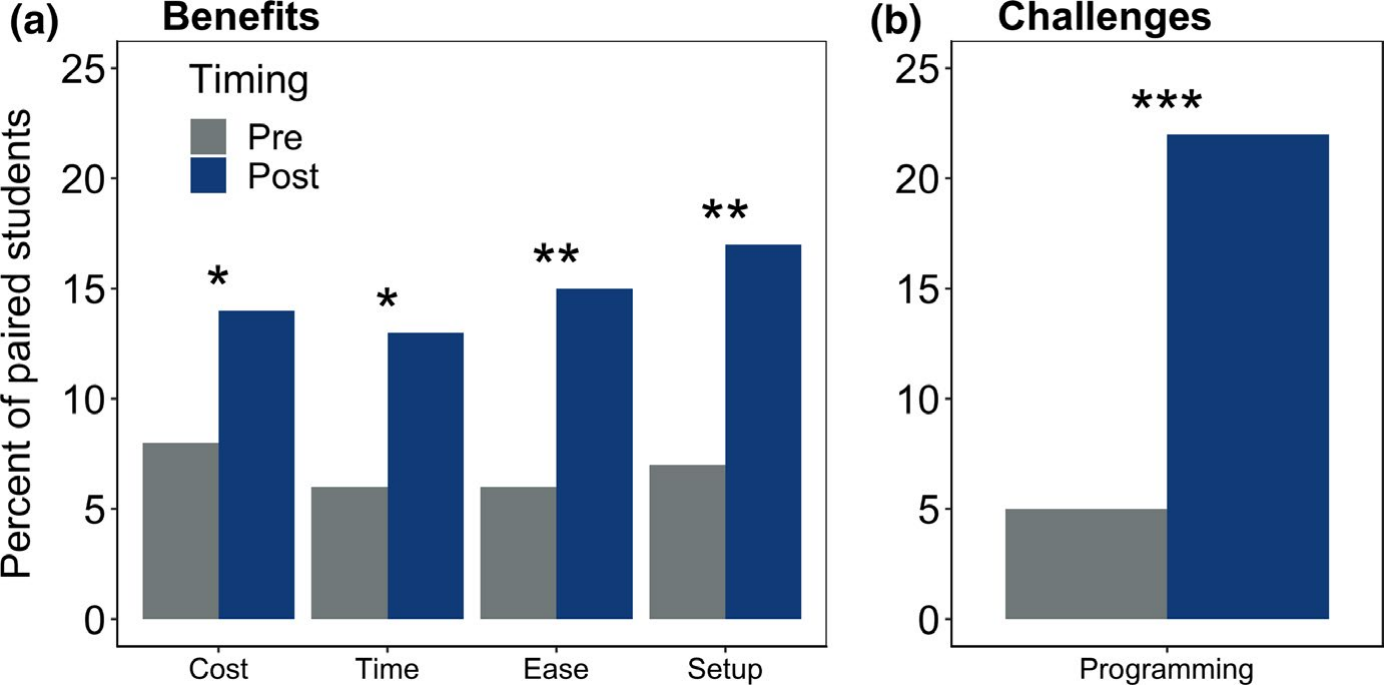
The percentage of students reporting potential benefits (a) of cost savings, time savings, ease of use, and model setup/manipulation; and challenges (b) of programming/coding using ecosystem models in paired premodule and postmodule qualitative assessment responses. Stars indicate statistical significance; ***p < .001; **p < .01; *p < .05

As a result of completing one module, students’ perceptions of benefits and challenges of working with models significantly changed (Table 4, Table S2). Students were significantly more likely to report in the postmodule assessment that the greatest benefits of models were their ease of use and the ability to set up a model to manipulate driver variables or parameters. For example, one student responded in the postmodule assessment, “Ecosystem models allow[s] you to adjust different variables within the model to see what other variables are affected by that change.” In addition, students were also significantly more likely to respond in the postmodule assessment that the benefits of ecosystem models included their low monetary cost and time savings. For example, one respondent noted that ecosystem models “Allow you to test hypotheses without having to spend the time and money it would take to test hypotheses in an actual lake,” while another noted, “You can run a lot of models quickly and get results faster than with an experiment” (Figure 4a).

**TABLE 4:** Differences in student responses of the benefits and challenges of using ecosystem models, and evidence of the use of systems thinking in their qualitative answers between pre‐ and postmodule assessments

Metric	Test statistic	Two‐tailed *p*‐value	*n*	Premodule (%)	Postmodule (%)	Effect size
Benefits of models						
Cost savings	12	**.039**	130	8	14	−0.18
Ease of use	42	**.008**	130	6	15	−0.23
Model setup/manipulation	60	**.007**	130	7	17	−0.24
Time savings	50	**.04**	130	6	13	−0.18
Challenges of models						
Programming/coding	39	**<.001**	107	5	22	−0.37
Any evidence of systems thinking						
Benefits of models	38	**.020**	76	2.6	9.2	0.19
Challenges of models	63	.484	70	5.0	7.1	−0.06

Note

Student responses to qualitative questions were binned for analysis. Test statistics and *p*‐values are for paired, two‐sided Wilcoxon signed‐rank tests. Note that only statistically significant (*α* = .05; bold) metrics are reported here for ecosystem modeling benefits and challenges; for full statistical results, see Table S2. Full statistical results for evidence of systems thinking are in Table S3.

Students were also more likely to say that programming/coding was a challenge of working with ecosystem models, suggesting that completing the module resulted in a greater understanding of the nuances and complexities of working with ecosystem models (Table 4, Figure 4b). For many students, this was their first experience working in R, resulting in multiple comments to the question about model challenges such as, “You have to be very careful with the modeling bec[ause] one period can make your code wrong.”

### 3.3 Question 2: How does using hands‐on Macrosystems EDDIE modeling activities affect students’ use of systems thinking?

**FIGURE 5:**
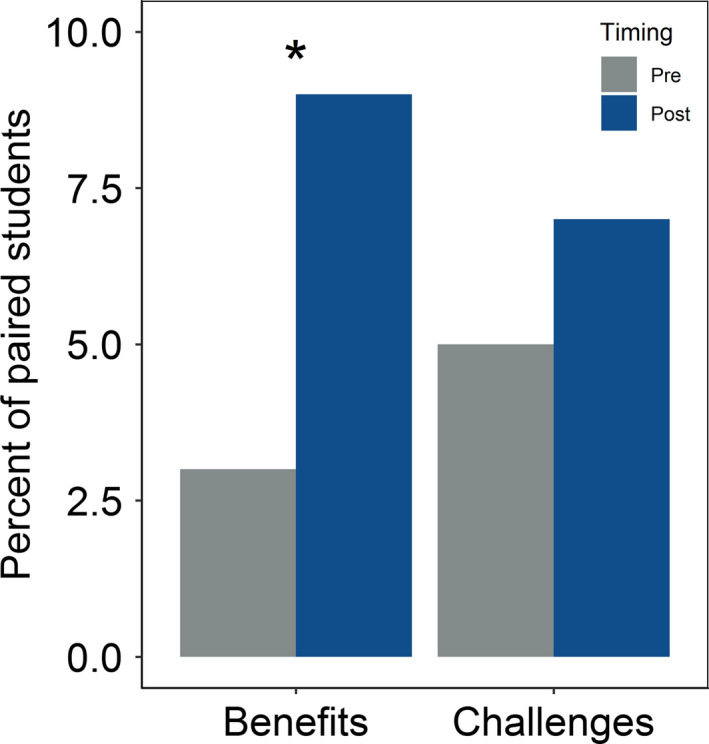
Evidence of systems thinking use from binned student free‐responses in pre‐ and postmodule assessments about the benefits and challenges of using ecosystem models. Star indicates statistical significance (*p < .05)

Students were significantly more likely to provide evidence of systems thinking in their qualitative responses to the question about benefits of ecosystem models after completing a module (*p* = .02; Table 4, Figure 5). In particular, students’ responses were more likely to describe how many variables may contribute to a given outcome and how new factors introduced to an ecosystem may influence model predictions, or how a change in part of an ecosystem could influence other parts of the ecosystem (Table S3). For example, one student noted in their postmodule response that two benefits of using ecosystem models included “Understanding which variables have the largest impact on a particular outcome” and “Understanding the full impacts that one variable or event could have on an ecosystem,” whereas another student responded that “Using ecosystem models allows you to adjust different variables within the model to see what other variables are affected by that change.” Similarly, another noted that a benefit of ecosystem models was “Making connections between elements that may not be evident, and observing how what may appear to be small changes can lead to large effects.” However, the overall increase in the number of student responses showing evidence of systems thinking was low (from 3% to 9% of respondents).

While evidence of systems thinking also increased in the qualitative responses to the question about the challenges of ecosystem models (from 5% to 7% of respondents), the change was not significant (*p* = .48; Table 4; Figure 5). Student responses to this question were focused primarily on the challenges inherent to setting up and running a complex ecosystem model: e.g., “[An ecosystem model] needs a full list of forcings and feedbacks to properly predict the system,” and “When you change one variable to see the outcome, you are assuming that all other variables are staying constant, when the reality is that they will not.”

## 4 DISCUSSION

Our study demonstrates that integrating a single 3‐hr Macrosystems EDDIE module into ecology courses may be a viable approach for instructors seeking to introduce their students to ecosystem modeling while also building their computational skills. Completing one Macrosystems EDDIE module significantly increased students' confidence and perceived proficiency working with models and, in some cases, increased students' use of a systems thinking perspective. These findings support previous work that found that including short‐term hands‐on data analysis activities in classrooms may have longer‐term benefits for students’ gains in computational literacy and systems thinking skills (Farrell & Carey, 2018; Gilbert et al., 2019; Monroe, Plate, & Colley, 2015), especially for undergraduate students (Carey & Gougis, 2017).

Student confidence and perceived proficiency working with simulation models and the General Lake Model significantly increased as a result of working with a Macrosystems EDDIE module (Table 3). Although small, these gains are important because students who lack confidence in their ability to use computational tools such as models may avoid engaging with such tool on their own (e.g., Baker, 2017; Farrell & Carey, 2018). Because Macrosystems EDDIE modules are designed to be used by first‐time modelers and R users, they can help build students’ perceived proficiency setting up and manipulating complex models across scaffolded activities. By building incrementally from simple to more complex tasks, module activities help foster a growth mindset in students (sensu O'Rourke, Haimovitz, Ballweber, Dweck, & Popovic, 2014; Elliot, Dweck, & Yeager, 2017; Limeri et al., 2020), whereby students are encouraged to embrace the challenge of trying something new and difficult. Such a shift in perceptions, for example, from a student saying they are “Not at all confident” to “Somewhat confident” about their ability to work with simulation models, may nudge them toward greater engagement long‐term in computational activities such as modeling.

We observed that students’ self‐reported premodule assessment of their proficiency and confidence influenced the magnitude of their postmodule gains (Figure 2), as the students who initially reported the least familiarity with ecosystem modeling exhibited the greatest gains in modeling proficiency, confidence, and future likely use. This pattern is likely due to both the ceiling effect, in that the students with the highest premodule scores are limited in their ability to show improvement on the 1‐to‐5 Likert scale (Vogt, 2005) and the Dunning‐Kruger effect, in which students often are overconfident in their initial abilities, and subsequent exposure results in lower postintervention than preintervention scores (Kruger & Dunning, 1999). This finding has specific relevance for teaching ecology students who are not familiar with modeling and suggests that integrating short ecosystem modeling activities into undergraduate‐level ecology curricula could increase undergraduate students’ confidence and ability to use ecosystem models long‐term. Specifically, it is possible that introducing undergraduates to ecosystem modeling may help overcome intimidation that might prevent them from using modeling in their careers or further study, though additional longitudinal data are needed to test this hypothesis.

Importantly, students' perceptions of ecosystem models significantly changed as a result of completing one Macrosystems EDDIE module. Students were significantly more likely to respond that a benefit of ecosystem models is their ease of use in the postmodule than premodule responses, suggesting that students were able to master the introductory activities presented in the modules. There was also a significant increase in the number of students in the postmodule assessment who described the benefits of manipulating models (e.g., varying meteorological driver data to explore different climate scenarios) as well as the cost and time savings of using models to conduct whole‐ecosystem experiments in comparison with empirical approaches. While we did not control for student experience level, many of the participating students were introduced to the R software environment for the first time as part of module activities, so it is not surprising that students were significantly more likely to identify programming/coding as a challenge of using ecosystem models in the postmodule assessment. Altogether, these responses suggest that Macrosystems EDDIE modules are able to provide students a realistic introduction to ecosystem modeling and an appreciation of the power of simulation models for exploring ecosystem dynamics.

Moreover, our study provides empirical evidence that short‐term Macrosystems EDDIE modeling activities may stimulate students’ use of systems thinking skills. Without being prompted, students independently identified multiple phenomena that relate to macrosystems‐scale behavior (e.g., multiple variables interacting to contribute to a given outcome; how a change in one part of an ecosystem could influence other parts of an ecosystem) in their qualitative item responses. Gaining an awareness of how individual components interact to affect a system may be a fundamental part of learning how to use ecosystem models, as all of the Macrosystems EDDIE modules asked students to develop or choose multiple model scenarios, run ecosystem models, analyze the model output, and interpret lake ecosystem responses to the scenarios. While our assessment did not directly ask students to self‐report their systems thinking ability and the proportion of these responses was low, these indirect metrics support previous work that introducing modeling in classrooms may enhance students’ use of systems thinking (e.g., Monroe et al., 2015).

Our study has particular relevance for teaching ecology students key concepts in macrosystems ecology, which is rooted in the ability to understand complex ecosystem processes that interact on multiple temporal and spatial scales (Heffernan et al., 2014). Our results suggest that using ecosystem modeling to teach macrosystems ecology in undergraduate and graduate classrooms may be a key tool for helping students grasp the complexities associated with thinking about processes that interact on multiple scales. These findings may be especially useful for improving aquatic ecology education, given the substantial environmental challenges facing freshwater and marine ecosystems that include phenomena occurring on multiple temporal and spatial scales (e.g., Heffernan et al., 2014; Rose et al., 2017). For example, in the “Cross‐Scale Interactions” Macrosystems EDDIE module, students analyze how climate change (which operates at a regional to global scale) may interact with land use change (which operates at a local scale) to increase the likelihood of phytoplankton blooms in lakes, depending on the lake's baseline water quality (Carey & Farrell, 2019).

This study was limited by our inability to control for differences in student experience level and other demographic differences, student:instructor ratio, and instructor experience with the R programming language. It is likely that student gains varied substantially among classrooms and modules, though we were unable to assess those differences given the wide range in the number of students per classroom that consented to participate in the study (Table 2). However, we note that the consistent responses across a relatively high total number of respondents (*n* = 277) support our conclusions that most students will exhibit gains in their ecosystem modeling proficiency, confidence, and future likely use as a result of completing a Macrosystems EDDIE module. Although we focused on self‐reported measures, previous work has demonstrated that self‐assessment can be a reliable metric of actual skill, especially for introductory students (reviewed by Ma & Winke, 2019 and references therein).

Importantly, our study demonstrates that Macrosystems EDDIE modules can be successfully integrated into a range of different ecology courses to teach students ecosystem modeling skills (Table 2). As the need for active learning teaching activities based in hands‐on scientific inquiry increases (Gilbert & Justí, 2016) as well as instructional materials that can be implemented remotely, the Macrosystems EDDIE curriculum provides a low‐stakes teaching approach for ecology instructors to introduce their students to modeling in a realistic context in as little as one laboratory period. Our assessment provides an important proof‐of‐concept that engaging students in even one short, hands‐on Macrosystems EDDIE ecosystem modeling module may stimulate students’ ability to think about how ecosystem functioning is shaped by interactions occurring on different spatial and temporal scales.

## CONFLICT OF INTEREST

The authors declare no conflicts of interest.

## AUTHOR CONTRIBUTION

**Cayelan C. Carey:** Conceptualization (lead); Data curation (supporting); Formal analysis (equal); Funding acquisition (lead); Investigation (lead); Methodology (lead); Resources (lead); Writing‐original draft (lead); Writing‐review & editing (lead). **Kaitlin J. Farrell:** Conceptualization (supporting); Data curation (lead); Formal analysis (lead); Investigation (equal); Methodology (equal); Resources (equal); Writing‐original draft (supporting); Writing‐review & editing (supporting). **Alexandria G. Hounshell:** Formal analysis (supporting); Investigation (supporting); Methodology (supporting); Resources (supporting); Writing‐review & editing (supporting). **Kristin O'Connell:** Data curation (equal); Formal analysis (equal); Investigation (supporting); Methodology (equal); Writing‐original draft (supporting); Writing‐review & editing (supporting).

## Supporting information

Supplementary Material

## Data Availability

All teaching module materials are available in the Environmental Data Initiative (EDI) repository (Carey et al., 2018; Carey & Farrell, 2019; Farrell & Carey, 2019). The human subjects assessment data presented in this manuscript cannot be publicly archived, per our Institutional Review Board protocol.
