# Supplementary material for: Macrosystems EDDIE teaching modules significantly increase ecology students' proficiency and confidence working with ecosystem models and use of systems thinking
Source: Ecol Evol. 2020 Oct 2;10(22):12515–27. doi: 10.1002/ece3.6757 (PMC7679539; doi:10.1002/ece3.6757)
Supplement: Supplementary file 1 — Supplementary Material [file ECE3-10-12515-s001.docx]

**Appendix S1**

**Table S1.** Multiple choice questions and associated Likert scales for student pre-module and post-module self-assessments as well as qualitative assessment questions used during this study.

| ***Multiple choice questions*** | | | | | |
| --- | --- | --- | --- | --- | --- |
|  | **1** | **2** | **3** | **4** | **5** |
| Using the scale below, how would you rank your proficiency with each of the following: *Simulation modeling; General Lake Model (GLM)* | No proficiency, not able to apply this tool to an assignment | Basic proficiency, able to handle simple applications of this tool to an assignment | Intermediate proficiency, able to apply this tool independently to many types of assignments | Advanced proficiency, able to apply this tool independently to nearly all types of assignments | Expert proficiency, able to apply this tool independently to all types of assignments and serve as a role model or coach others |
| Using the scale below, how would you rank your confidence level in working with each of the following: *Simulation modeling; General Lake Model (GLM)* | Not at all confident | Somewhat confident | Moderately confident | Very confident | Completely confident |
| Using the scale below, how likely are you to use the following tools in your future studies or research? *Simulation modeling; General Lake Model (GLM)* | Extremely unlikely | Unlikely | Neutral | Likely | Extremely likely |
| Please select the statement below that *best* describes your current knowledge of ecosystem simulation modeling: | Not at all familiar, I have never heard of ecosystem simulation modeling | Slightly familiar, I have heard of ecosystem simulation modeling, but cannot elaborate | Somewhat familiar, I could explain a little about ecosystem simulation modeling | Moderately familiar, I could explain quite a bit about ecosystem simulation modeling | Extremely familiar, I could explain and instruct others about ecosystem simulation modeling |
| ***Qualitative free-response questions*** | | | | | |
| 1. What do you think are the three greatest benefits of using ecosystem models? | | | | | |
| 1. What do you think are the three greatest challenges for using ecosystem models? | | | | | |

**Appendix S1. Detailed methods of the qualitative assessment analysis.**

During phase I, two Macrosystems EDDIE coordinators and two independent SERC evaluators reviewed student responses to the two qualitative assessment questions and recorded emerging themes. The coordinators and evaluators developed separate preliminary codebooks from their initial observations, which were then compared and refined. After developing the initial codebook, one coordinator and both evaluators completed two iterations of independent coding, comparing codes, resolving coding disagreements, and refining the codebook to better characterize relevant thematic bins of student responses. The initial set of coded responses included ~10% of the data selected using a random number generator, and ~8% of those responses were re-coded after codebook refinement. At the end of this procedure, the evaluators refined and pared-down the codebook by removing all unused thematic bins and more explicitly characterizing each remaining bin. The final codebook thematic bins are listed in Table A2.

During phase II, student responses were coded by the two evaluators using the updated codebook. During the coding, all identifying student, course, and survey (pre- vs. post-module) information was hidden, and responses were randomly-ordered based on a random number generator. The first 50 responses (~10% of the total pre-/post- responses) were independently coded by the two evaluators. Upon showing agreement in coding for those 50 responses, the evaluators split the remaining responses in half, with five responses in common to check agreement. The result of this two-phase process was a database of student responses coded by the presence or absence of each of the thematic bins in their responses to the two qualitative questions (Table A2). Wilcoxon signed-rank tests of paired student pre- and post-module responses were then conducted for each of the thematic bins as described above.

Finally, the same responses to the two qualitative assessment questions were reviewed for evidence of systems thinking. All identifying student, course, and survey (pre- or post-module) information was hidden. To evaluate if there was evidence of systems thinking, the two evaluators independently coded a randomly-selected ~10% of the student responses using an *a priori* codebook based on the four rubric categories developed by Iverson et al. (2019). Codes were then compared, reconciled, and the codebook was refined to include an additional category to capture evidence of systems thinking: 5) Student correctly describes that many variables may contribute to a given outcome or new aspects introduced in a system may influence predictions. The evaluators then independently coded all student responses, compared codes, and reconciled differences from both qualitative questions. The final database of student responses included the five individual categories as well as a collapsed category that summed all of the individual categories, indicating the presence or absence of any evidence of systems thinking (Table A3).

**Table S2.** Full statistical results for differences in student pre-module and post-module assessment responses of the benefits and challenges of using ecosystem models. The thematic bins show the full list of codebook categories that were evaluated to be either present or absent in the student responses. Student responses to the qualitative questions were binned for analysis, as described in the text. Test statistics and p-values are for paired, two-sided Wilcoxon signed-rank tests. Significant p-values are in bold.

| **Thematic bin** | **Test statistic** | **Two-tailed**  **p*-*value** | **Pre-module**  **(%)** | **Post-module**  **(%)** | **Effect**  **size** |
| --- | --- | --- | --- | --- | --- |
| *Benefits of models* (n = 130) |  |  |  |  |  |
| Accuracy | 12 | 0.78 | 3 | 4 | -0.02 |
| Cost savings | 12 | **0.039** | 8 | 14 | -0.18 |
| Ease of use | 42 | **0.008** | 6 | 15 | -0.23 |
| Many scenarios/simulations | 45 | 0.62 | 6 | 8 | -0.04 |
| Model setup/manipulation | 60 | **0.007** | 7 | 17 | -0.24 |
| No field or lab work required | 92 | 0.21 | 11 | 15 | -0.11 |
| Prediction/forecasting | 484 | 0.88 | 56 | 55 | -0.01 |
| Science communication | 17.5 | 0.13 | 5 | 2 | -0.13 |
| Societal uses | 30 | 0.80 | 6 | 7 | -0.02 |
| Time savings | 50 | **0.04** | 6 | 13 | -0.18 |
| Understand an ecosystem | 374 | 0.06 | 23 | 15 | -0.17 |
| Visualization | 95 | 0.66 | 15 | 10 | -0.04 |
| *Challenges of models* (n = 107) |  |  |  |  |  |
| Challenging to use | 99 | 0.24 | 11 | 7 | -0.11 |
| Communication of results | 0 | 0.07 | 0 | 4 | -0.17 |
| Cost savings | 8 | 0.30 | 3 | 6 | -0.1 |
| Data (quality, quantity, or management) | 451.5 | 1.00 | 38 | 38 | 0 |
| Human errors | 12 | 0.78 | 7 | 7 | -0.03 |
| Limitations of models | 279 | 0.28 | 62 | 56 | -0.1 |
| Programming & coding | 39 | **<0.001** | 5 | 22 | -0.37 |
| Software, technology, and computers | 63 | 0.08 | 6 | 13 | -0.17 |
| Teaching/training needed | 0 | 0.15 | 3 | 6 | -0.14 |
| Time savings | 12 | 0.23 | 5 | 2 | -0.12 |

**Table S3.** Full statistical results for differences in student pre-module and post-module assessments indicating evidence of use of systems thinking in responses to qualitative questions on the benefits and challenges of using ecosystem models. Pre-module and post-module counts represent the number of students that exhibited evidence of systems thinking for each criterion (modified from the rubric of Iverson et al. 2019); note that “Any evidence present” summarizes all of the systems thinking criteria, and represents any evidence of the presence of systems thinking in student responses. Test statistics and p-values are for paired, two-sided Wilcoxon signed-rank tests; tests were not run for items with zero responses.

| **Criterion** | **Test statistic** | **Two-tailed**  **p-value** | **Pre-module**  ***n*** | **Post-module**  ***n*** | **Effect**  **size** |
| --- | --- | --- | --- | --- | --- |
| *Benefits of models* (n = 76) |  |  |  |  |  |
| Any evidence present | 38 | **0.020** | 4 | 14 | 0.19 |
| Describes how a change in one part of the system, in turn, alters other parts of the system | 8 | 0.299 | 2 | 5 | 0.08 |
| Describes how an effect can be influenced by multiple causal factors | 0 | 1.000 | 0 | 1 | 0.00 |
| Describes that many variables may contribute to a given outcome or new aspects introduced in a system may influence predictions | 11 | 0.066 | 2 | 8 | 0.15 |
| Explains how parts of the system interact using systems concepts such as feedbacks, equilibrium, rates, etc. | - | - | 0 | 0 | - |
| Identifies and describes a real-world system including its parts | - | - | 0 | 0 | - |
| *Challenges of models* (n = 70) |  |  |  |  |  |
| Any evidence present | 63 | 0.484 | 7 | 10 | 0.06 |
| Describes how a change in one part of the system, in turn, alters other parts of the system | 4 | 0.773 | 2 | 1 | -0.02 |
| Describes how an effect can be influenced by multiple causal factors | - | - | 0 | 0 | - |
| Describes that many variables may contribute to a given outcome or new aspects introduced in a system may influence predictions | 26 | 0.267 | 4 | 8 | 0.09 |
| Explains how parts of the system interact using systems concepts such as feedbacks, equilibrium, rates, etc. | 1.5 | 1.000 | 1 | 1 | 0.00 |
| Identifies and describes a real-world system including its parts | - | - | 0 | 0 | - |
